# Reliability and validity of the problematic TikTok Use Scale among the general population

**DOI:** 10.3389/fpsyt.2023.1068431

**Published:** 2023-03-28

**Authors:** Aykut Günlü, Tuncay Oral, Soyoung Yoo, Seockhoon Chung

**Affiliations:** ^1^Department of Child Care and Youth Services, Pamukkale University, Denizli, Türkiye; ^2^Department of Convergence Medicine, Asan Medical Center, University of Ulsan College of Medicine, Seoul, Republic of Korea; ^3^Department of Psychiatry, Asan Medical Center, University of Ulsan College of Medicine, Seoul, Republic of Korea

**Keywords:** validation, reliability, social media, psychology, TikTok, problematic use

## Abstract

**Introduction:**

This study aims to provide a scale for measuring problematic TikTok use levels by adapting items from the Instagram Addiction Scale.

**Methods:**

The 372 participants were determined by a convenience sampling method, and data were collected through Google online forms. Exploratory Factor Analysis (EFA) and Confirmatory Factor Analysis (CFA) were performed for construct validity and criterion-related validity analysis. Criterion-related validity for the Problematic TikTok Use Scale (PTTUS) was tested using correlation analysis between the Bergen Social Media Addiction Scale and Social Media Use Disorder Scale.

**Results:**

EFA indicated that a three-factor structure should be formed. The first factor is the sub-dimension of obsession and consists of 4 items, the second factor is the escapism sub-dimension and consists of 6 items, and the third factor is the lack of control sub-dimension and consists of 6 items. The model fit for adapting the PTTUS into Turkish was examined with first-level CFA, χ2/sd, RMSEA, CFI, GFI, AGFI, and SRMR, the obtained values show that the three-factor structure of the scale provides acceptable fit. Reliability analyses showed that Cronbach’s alpha internal consistency reliability coefficient ranged from 0.83 to 0.90; McDonald’s Omega reliability values was 0.84 to 0.90, and test–retest correlation coefficient ranged from 0.68 to 0.73, indicating sufficient internal consistency and test–retest reliability.

**Conclusion:**

Based on this information, PTTUS is a measurement tool with sufficient psychometric properties that can be applied to determine individuals’ levels of problematic TikTok use.

## Introduction

The first smartphones began to enter people’s lives in the early 2000s, offering ease of use without limitations of time and place. They have since become a necessity in many areas of life; smartphone use has become widespread, and its importance has gradually increased. Internet usage on smartphones now exceeds the rate of internet usage on other devices, such as computers and tablets, reaching 95.5%. Social media is the most common type of smartphone internet use. Among social media applications, YouTube (23.7%) is the most popular, followed by Facebook (23.6%) and TikTok (19.6%) in terms of time spent. There has been a significant increase in social media use, especially during the COVID-19 (Coranavirus) Pandemic (1).

Behavioral addiction is a person’s actions and activities that cause physiological, psychological and social problems and continue to be done uncontrollably despite the person’s desire to quit, thus considering this behavior as unimportant and continuing to do it even if it harms herself/himself and her/him environment. Behavioral addiction according to DSM-5; It has features such as being overly preoccupied with behavior, decreased ability to control behavior, developing tolerance for behavioral, exhibiting excessive negative emotions when trying to avoid behavior, and causing negative psychological problems such as stress and depression.

TikTok was launched in 2016 by the China-based company ByteDance, under the name Musical.ly; it was renamed “TikTok” a year later (2). The TikTok application is used by downloading it to a smartphone and allows users to record videos of less than 3 min, which users can edit themselves (3). Some features include adding audio and images, making live broadcasts, and earning a certain amount of income based on users’ number of followers. It differs from other social media platforms in that it can add audio and images to videos, produce content in line with followers’ interest with short videos, enable more interaction, and involve users in an interactive process. TikTok allows people to both entertain and earn income while producing content and trying to attract followers’ attention, which increases its use.

TikTok was downloaded more than 2 billion times in 2021, and most users are adolescents and young adults (16–35 years old) (4). According to the statistical data, 68.97% of TikTok users are under the age of 24, and 73.69% are under the age of 30 (5). Social media defines the phenomena it creates with the name of the social media network. For example, someone who is famous on YouTube is called a YouTuber, someone who is famous on Instagram is an Instagrammer, and someone famous on TikTok is defined as TikToker. TikTokers prepare and share content for reasons such as social acceptance, feeling comfortable, and satisfaction (6), which contributes to increasing TikTok usage.

It has been reported that social media have negative physical, psychological, emotional, and social effects on individuals (7–10). Previous studies have investigated and defined Facebook (11–13), Twitter, and YouTube (14, 15) addiction. Social media addiction is accepted as a subtype of internet addiction, which is one of the behavioral addiction types (8, 10, 12). Some recent studies emphasize that many symptoms seen in internet addiction are also included in social media addiction. In addition, in recent years, social media, which has increased its use, can make individuals more addicted. In previous years, there are many studies on social media addictions such as Facebook Addiction, Twitter Addiction, Youtube Addiction, and what these addiction types are has been defined. For example, there is the Social Media Addiction (SMD) scale, which was developed by Tutkun-Ünal (16) and whose reliability and validity studies were conducted, in order to detect social media addiction. This scale was developed to measure the social media addictions of university students in various ways such as gender, age, class level, applications used, school where they study, social media usage tools, people they live with, and duration of use of social networks. Another social media addiction scale was adapted into Turkish by Demirci (17). The scale aims to determine the mental pursuit, mood change, tolerance, deprivation, conflict and unsuccessful attempts of individuals to use social media. There are also social media addiction scales developed on different samples (18, 19). Scale adaptation studies were also carried out in order to measure Facebook addiction, another social media platform. The “Facebook Addiction Scale” was developed by Kimberly Young in 1998 to measure internet addiction and was adapted to Facebook by Çam (20) and translated into Turkish. There is also a facebook addiction scale developed by Turkyilmaz (13) and Akın et al. (11). As a result, it is seen that a wide variety of scales have been developed or adapted to determine the sub-types of technology or the levels of addiction in various social media platforms. However, it is stated that the TikTok application, which has become widespread in recent years, is now at the level of addiction. In the literature review, no scale was found to measure problematic TikTok use. In this respect, the absence of scale for the problematic TikTok use emerges as an important deficiency in the field.

Today, TikTok, one of the social media platforms today, has become an problematic due to its increasing usage rate. Today, problematic TikTok use has also become a concern, and can be defined as spending excessive time on one’s own page to increase the number of followers; increasing the amount of time spent using the application day by day; the inability to control the time spent; and eventually getting bored of real life and coming to seeing one’s virtual identity as real and arranging one’s lifestyle accordingly. Moreover, individuals who are addicted may feel tense, restless, stressed, and lonely when they cannot use it.

India has the highest number of TikTok users, followed by the United States and Turkey. The average monthly TikTok usage time in Turkey is 18.8 h (1). Since problematic TikTok use is a relatively new phenomenon, there is comparatively little research investigating it, and it can be difficult to determine problematic TikTok use. While there are scales measuring other types of social media addiction in the literature, there is no scale for specifically measuring problematic TikTok use. Thus, this study aims to provide a scale for measuring problematic TikTok use.

## Method

Before starting reliability and validity studies of the new scale, the necessary permissions were obtained from the author of the scale *via* email. And then the development process of the scale, necessary permissions were obtained from Pamukkale University Social and Human Sciences Ethics Committee in accordance with the decision of Document Date: 31.05.2022 and Number of Documents: E-93803232-622.02-211,894. After the items of the original scale were adapted for the PTTUS, they were sent to three experts working on the subject for content validity. In line with the suggestions from each expert, the item list of the scale was finalized and a pilot application was made. This indicated that the items of the scale worked well, and the trial phase began. Data were obtained *via* Google forms.

### Participants and procedures

Participants were 500 Turkish adults who were determined by convenience sampling. Data were collected using Google forms. Forms were distributed over the internet to Pamukkale University students, their relatives, and university staff. Responses with missing or extreme data were excluded from the analysis, leaving a final sample of 372 (74.4%). The construct validity of the scale was examined by confirmatory analysis. For factor analysis, according to Tabachnick and Fidell (21), 300 people in the research group is considered good, and 500 people is considered very good. In this respect, it can be said that the student group in which the studies are carried out is sufficient in terms of the number of personnel required by statistical analysis. The first page of the form included the purpose of the study, the ages and genders of the researchers, and the voluntary participant consent form. The second page of the form contained the scale items, which were scored using Likert-type scales. Participants knew about and used TikTok, and came from 52 cities in 7 regions of Turkey.

### Measures

All rating scales were delivered to the participants and all data were collected within 2 weeks. In addition, the scale was sent to the same participants after a two-week break for test–retest reliability. Participants were asked to add their email addresses before submitting their questionnaires (used for informational purposes for the study only, and the information was deleted immediately after the analysis) for use in the test–retest analysis. A total of 215 participants; therefore, the test–retest was carried out with data from 215 participants.

#### The original form of the Problematic TikTok Use Scale

The PTTUS was developed by adapting D’Souza et al. (22) Instagram Addiction Scale, whose psychometric properties were determined by Kavaklı and İnan (23) (Supplementary Material S1). Permission was obtained from both the authors of the scale and the social and human sciences ethics committee of the university before adapting the scale. After the items of the Instagram Addiction Scale were adapted for the PTTUS, expert opinion was sought for the scale items and suggested corrections were made. The original Instagram Addiction Scale consisted of 21 items, 16 of which were adapted for the PTTUS. Originally, a 21-item PTTUS was presented to participants. However, as a result of EFA, five items whose factor load was not sufficient to be included in any factor were removed from the scale. Items were scored using a 5-point Likert-type scale (1 = *never* and 5 = *always*). One sample item is “I often upload videos to TikTok.” Higher scores indicate higher levels of problematic TikTok use. In the current study, the Cronbach’s Alpha reliability of the scale was 0.90; the Cronbach’s Alpha reliability of the sub-dimensions; 0.84 for obsession sub-dimension; 0.90 for escapism sub-dimension and 0.85 for lack of control sub-dimension. In the current study, the McDonald’s Omega reliability of the scale was 0.90; the McDonald’s Omega reliability of the sub-dimensions; 0.84 for obsession sub-dimension; 0.90 for escapism sub-dimension and 0.85 for lack of control sub-dimension.

#### Bergen Social Media Addiction scale

The BSMAS scale was developed by Schou Andreassen et al. (24) and adapted into Turkish by Demirci (17). The scale consists of six items measuring mental exertion, mood change, tolerance, withdrawal, conflict, and unsuccessful attempts to quit. Items are scored using a 5-point Likert-type scale; higher scores reflect higher dependence on social network sites. Total scores range from 6 to 30. In the adaptation study, the Cronbach alpha internal consistency reliability coefficient of the scale was found 0.83. In this current research, the Cronbach alpha internal consistency reliability coefficient of the scale was found 0.857, and McDonald’s Omega reliability value was calculated as 0.860.

#### Social Media Disorder scale

Developed by Van den Eijnden et al. (25) to measure individuals’ social media addiction levels, the SMD scale was adapted into Turkish by Sarıçam and Adam-Karduz (26) using a nine-item form. Each item measures a different sub-dimension (occupation, endurance, deprivation, insistence, escape, problems, deception, displacement, conflict). In the present study, the internal consistency coefficient of the scale was 0.75 and Cronbach’s alpha reliability coefficient was 0.82. In the adaptation study, the Cronbach alpha internal consistency reliability coefficient of the scale was found 0.75. In this current research, the Cronbach alpha internal consistency reliability coefficient of this scale was found 0.879, and McDonald’s Omega reliability value was calculated as 0.883.

### Statistical analysis

We examined the construct validity and reliability of the Turkish version of the PTTUS. Normality assumption was tested based on skewness and kurtosis of each item. To check the sampling adequacy and data suitability, the Kaiser–Meyer–Olkin (KMO) value and Bartlett’s test of sphericity were checked. The Exploratory Factor Analysis (EFA) with Varimax technique was used to determine the factor structure of the PTTUS. Factors with an eigenvalue above 1 were defined as acceptable. Confirmatory Factor Analysis (CFA) with the diagonally weighted least squares method was carried to check the factor structure of the PTTUS, and a satisfactory model fit for the model was defined by a standardized root-mean square residual (SRMR) value ≤0.05, root-mean-square-error of approximation (RMSEA) value ≤0.10, and comparative fit index (CFI) and Tucker–Lewis index (TLI) values ≥0.90. The Cronbach’s alpha method was preferred in the reliability analysis of the scale. The BSMAS and SMD scale were used for criterion-related validity. The receiver operating characteristic (ROC) analysis was performed to explore the appropriate cut-off score of the PTTUS on accordance with addiction (excessive or problematic use of social media and spending at least 8.5 to 21.5 h a week online). Validity and reliability analyses were conducted using the SPSS 22 and AMOS 20 package programs.

## Results

All 500 participants who knew about and used TikTok from 52 cities in 7 regions of Turkey responded to the survey. A total of 201 (54.04%) participants were female, and the age range was 18–40 (x̄ = 24.35; sd: 2.3).

### Analysis of exploratory factors

In the EFA, Kaiser-Meyer-Olkin (KMO) and Bartlett’s Sphericity tests were performed to test the suitability of the obtained data for factor analysis. Considering the analysis results, the KMO was 0.87 and the Approximate Chi-Square (**χ**^**2**^) result was 2918.35 (*p* < 0.001). Since the KMO was higher than 0.60 and the Barlett’s Sphericity test was significant, the dataset in the research group was considered to be suitable for factor analysis. (27) Findings related to the EFA were tested in the validity analysis. Varimax technique was used to determine the factor structure of the scale, so the factor number of the scale, the load values of each item, and the correlation of the items with the whole scale (Item-total correlation) were determined. Factors with an eigenvalue above 1 were accepted as the basis in the analysis (21).

In the adapted scale, a 3-dimensional structure with the sub-dimensions of obsession, escapism, and lack of control was obtained. Items 1–4 are in the first sub-dimension, items 5–10 are scored in the second sub-dimension, and items 11–16 are scored in the third sub-dimension. The eigenvalues and explanatory variances of the factor structures obtained as a result of the EFA are given in Table 1.

**Table 1:** Ratios of variance explained by eigenvalues obtained as a result of exploratory factor analysis.

	Initial eigenvalues			Rotation sums of squared loadings		
Factor	Total	% of variance	Cumulative (%)	Total	% of variance	Cumulative (%)
1	6.10	38.14	38.14	3.88	24.23	24.23
2	2.13	13.31	51.45	3.50	21.84	46.07
3	1.85	11.54	62.99	2.71	16.92	62.99

As a result of the EFA, the obtained three-factor structure explained 62.99% of the total variance. When the explanation rates of the factors were examined, factor 1 explained 24.23% of the total variance, factor 2 explained 21.84%, and factor 3 explained 16.92%.

### Analysis of confirmatory factors

**Figure 1:**
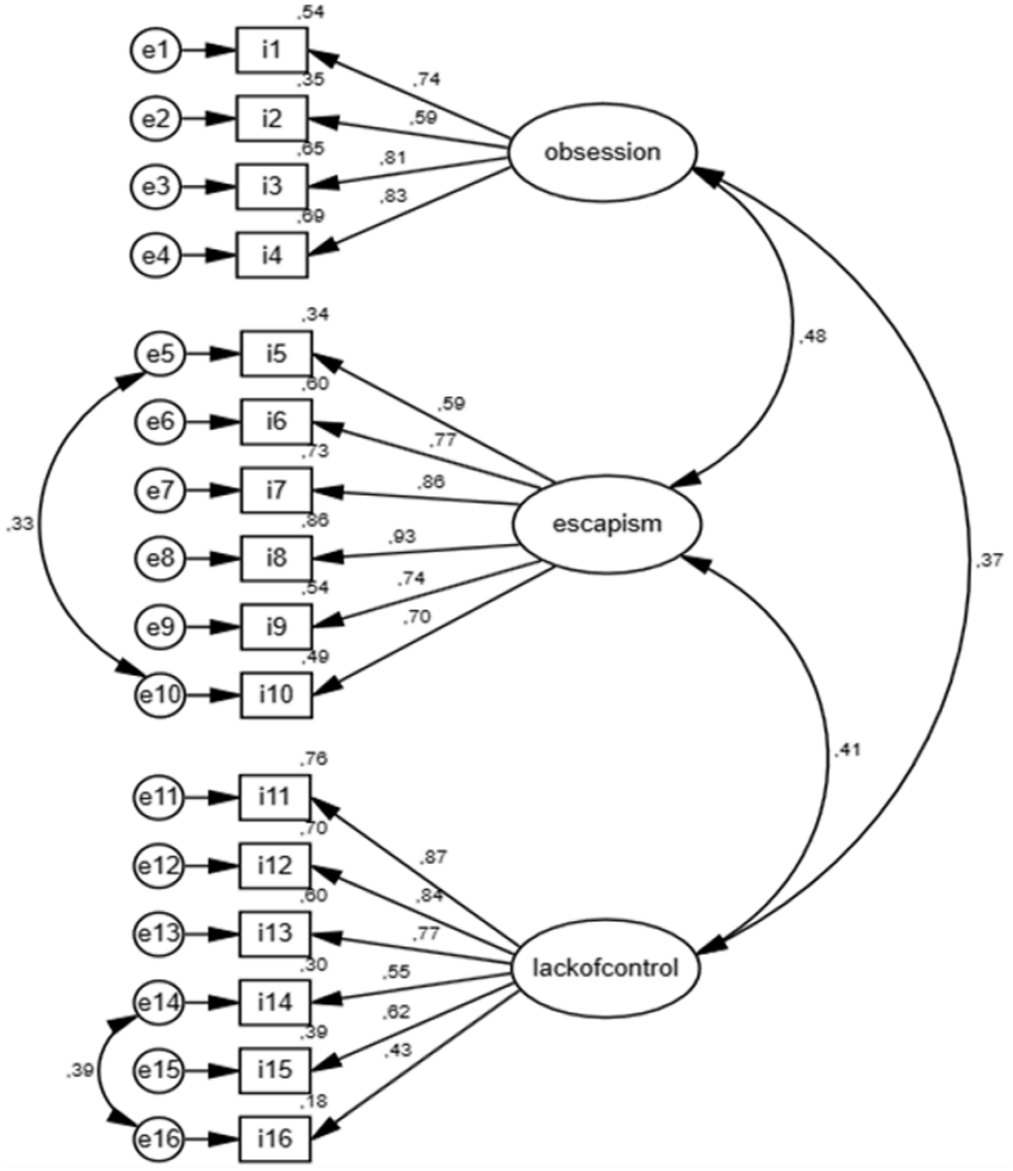
Path diagram and factor loads of the Problematic TikTok Use Scale.

CFA was performed to determine the scale’s structure (28, 29). Fit indices frequently used in CFA include Chi-square fit (χ2) and ratio of Chi-square to degrees of freedom (χ2/sd), Root Mean Square Errors of Approximation (RMSEA), Adjusted Goodness-of-Fit Index (AGFI), Comparative Fit Index (CFI), Goodness-of-Fit Index (GFI), and Standardized Root-Square Means (SRMR) (30). The data of the CFA performed to determine the construct validity of the PTTUS are presented in Table 2 and Figure 1. Item-total correlation and Cronbach’s alpha if item deleted reliability coefficient for each item are also shown (Table 2).

**Table 2:** Item factor loads for the Prpblematic TikTok Use Scale.

Item No.	Factor 1	Factor 2	Factor 3	Item-total correlation	Cronbach’s alpha if item deleted	McDonald’s Omega	Mean	SD
Item 1	0.74			0.45	0.90	0.90	1.60	0.90
Item 2	0.59			0.42	0.90	0.90	1.54	0.87
Item 3	0.81			0.56	0.89	0.90	1.59	1.06
Item 4	0.83			0.55	0.90	0.90	2.10	1.39
Item 5		0.59		0.66	0.89	0.90	2.25	1.30
Item 6		0.77		0.64	0.89	0.89	2.33	1.30
Item 7		0.86		0.65	0.89	0.89	2.85	1.38
Item 8		0.93		0.70	0.89	0.89	2.55	1.34
Item 9		0.74		0.64	0.89	0.89	2.04	1.26
Item 10		0.70		0.67	0.89	0.89	2.77	1.44
Item 11			0.87	0.53	0.90	0.90	1.53	0.96
Item 12			0.84	0.48	0.90	0.90	1.45	0.89
Item 13			0.77	0.57	0.89	0.90	1.55	0.93
Item 14			0.55	0.55	0.89	0.90	1.70	1.05
Item 15			0.62	0.62	0.89	0.89	0.51	0.97
Item 16			0.43	0.40	0.90	0.90	1.89	1.17

Looking at the scale’s sub-dimensions, factor 1 is the obsession sub-dimension comprising items 1–4; factor 2 is the escapism sub-dimension comprising items 5–10; and factor 3 is the lack of control sub-dimension comprising items 11–16. The item factor load distributions for the overall scale are shown in Table 2. To determine the item validity of the PTTUS, the item-total correlation results were examined. It is seen that the item-total correlation values vary between 0.40 and 0.70. Considering that items with an item-total correlation value of 0.30 and above are considered sufficient in terms of distinguishing the quality to be measured (27), all the items in the scale are sufficiently related to the scale’s total score and the scale item validity is ensured.

### Confirmatory factor analysis

While adapting PTTUS, model fit was examined using first-level CFA. The fit index values of the PTTUS were calculated χ2 = 1036.86, *p* < 0.01, χ^2^/sd = 4.19, RMSEA = 0.10, CFI = 0.88, GFI = 0.86, AGFI = 0.81, SRMR = 0.09 as before covariance. After was made covariance the fit indices of the PTTUS seem to be sufficient [CFA: χ2 = 1036.86, *p* < 0.01, χ^2^/sd = 4.14, RMSEA = 0.08, CFI = 0.95, GFI = 0.89, AGFI = 0.85, SRMR = 0.08]. Considering the statistical values of fit, a value of χ2/sd below 5 indicates acceptable fit, a RMSEA value between 0.00 and 0.05 indicates good fit, and a value between 0.05 and 0.08 indicates acceptable fit (31, 32). The factor loads of the items in the scale range between 0.44 and 0.93. The analysis of the first-level CFA is presented in Table 3.

**Table 3:** Model fit indices for the Problematic TikTok Use Scale.

	χ^2^/sd	RMSEA	CFI	GFI	AGFI	SRMR
Good fit values	<3	0.00–0.05	≥0.97	≥0.90	≥0.90	≤0.05
Acceptable fit	≤3–5	0.05–0.08	≥ 0.95	0.89–0.85	0.89–0.85	0.05–0.08
Model fit indices	4.14	0.08	0.95	0.89	0.85	0.08
Model fit indices for male	2.69	0.08	0.93	0.88	0.85	0.08
Model fit indices for female	2.78	0.08	0.95	0.87	0.85	0.08

### Criterion-related validity

For the criterion-related validity, correlations between the BSMAS and SMD scale were calculated and the analysis results are given in Supplementary Table S1.

When the relationships between PTTUS and BSMAS and SMD scale were examined, the following positive and significant relationships were found: between the PTTUS total score and BSMAS (*r* = 0.56, *p* < 0.01) and SMD scale (*r* = 0.49, *p* < 0.01); between the obsession sub-dimension and BSMAS (*r* = 0.35, *p* < 0.01) and SMD scale (*r* = 0.30, *p* < 0.01); between the escapism sub-dimension and BSMAS (*r* = 0.52, *p* < 0.01) and SMD scale (*r* = 0.39, *p* < 0.01); between the lack of control dimension and BSMAS (*r* = 0.45, *p* < 0.01) and SMD scale (*r* = 0.49, *p* < 0.01). Considering the analysis results align with the theoretical framework, it can be said that the PTTUS has criterion-related validity.

### Reliability analysis

In this study, Cronbach’s alpha internal consistency coefficient and test–retest reliability analysis were performed at two-week intervals to determine the reliability of the scale; the findings are presented in Supplementary Table S2.

Cronbach’s alpha internal consistency coefficient and test–retest reliability analysis were used to determine the reliability of the PTTUS. The analysis showed Cronbach’s alpha reliability coefficient of the total scale was calculated as 0.90. Reliability was 0.83, 0.90, and 0.85 for the obsession, escapism, and lack of control sub-dimensions, respectively. McDonald’s Omega reliability coefficient of the total scale was calculated as 0.90. Reliability for the obsession sub-dimension of the scale was 0.84; for the escapism sub-dimension, the reliability was 0.90; for the lack of control sub-dimension, the reliability was calculated as 0.85. The test–retest reliability analysis coefficients were 0.73 for the total scale, 0.68 for obsession, 0.68 for escapism, and 0.70 for lack of control. Considering that reliability coefficients of 0.70 and above are considered reliable in the scale adaptation process (33), it can be said that the internal consistency and test–retest reliability coefficients of the PTTUS are sufficient.

### The receiver operating characteristic

We performed the analysis of ROC and the area under the curve (AUC) for determining the PTTUS. Supplementary Table S3 shows the analysis of ROC with the parameters required. The cut-off value obtained for the PTTUS was ≥31.5, with sensitivity and specificity percentages of 88.9 and 37.5%, respectively. Subjects are diagnosed as experiencing problematic TikTok use if their score is ≥32.

## Discussion

The current study aimed to develop the PTTUS. For this purpose, the items of the Instagram Addiction Scale were adapted for Problematic TikTok Use. The TikTok application is closer to the Instagram application than the Facebook application due to its intended use and the developable content it allows. However, it is said that Instagram and TikTok applications tend to be used mostly on smartphones. In addition, it is seen that the use of Facebook application tends towards a more restricted age group. In addition, it has been determined that individuals use more interactive and instant sharing applications on social media (34). However, as the items of the Instagram addiction scale were evaluated to be more useful for measuring problematic TikTok use, it was deemed appropriate to use the items of the Instagram Addiction Scale. The Instagram addiction scale is a more inclusive scale since the number of items is 16. At the same time, the Instagram addiction scale was preferred because it is a more up-to-date scale. Language validity and content validity were performed for the obtained scale. In addition, EFA and CFA were performed for construct validity and criterion-related validity analysis was calculated. Cronbach’s alpha internal consistency, McDonald’s Omega value and test–retest coefficients were performed at two-week intervals to test the scale’s reliability.

The language validity of the scale was ensured in line with expert opinions obtained during the adaptation of the original scale items to TikTok. Before the EFA and CFA analysis, the dataset’s suitability for factor analysis was tested using KMO and Barlett tests. The dataset was considered suitable for factor analysis if the KMO was higher than 0.60 and the Barlett Sphericity test was significant. (27, 35) EFA and CFA showed that a three-factor structure consisting of 16 items explained 62.99% of the total variance, and the structure of the scale was confirmed. It can be said that PTTUS has a sufficient total variance explanation rate.

The CFA indicated that a three-factor structure is formed. The first factor comprises the obsession sub-dimension and consists of four items; the second factor is the escapism sub-dimension and consists of six items, and the third factor is the lack of control sub-dimension and consists of six items. The item factor load distributions of the scale showed load values ranging between 0.43 and 0.93. Considering that the item factor load value should be >0.32 (21) and if an item is included in more than one factor, there should be a difference of at least 0.10 in the item load between the factors (36), it can be said that the item factor load values of the three-factor PTTUS are sufficient. The model fit for adapting the PTTUS into Turkish was examined with the first-level CFA. χ2/sd, RMSEA, CFI, GFI, AGFI and SRMR values obtained as a result show that the three-factor structure of the scale provides acceptable fit.

In the study, correlations between BSMAS and SMD scale were calculated for criterion-related validity. The results showed that the total score and sub-dimensions of PTTUS had significant relationships with BSMAS and SMD scale. Considering the analysis results and the theoretical framework of PTTUS, it can be said that PTTUS has criterion-related validity. Corrected item-total correlations ranged from 0.40 to 0.70. The Cronbach’s alpha internal consistency and test–retest analyses were used to determine the scale’s reliability. Cronbach’s alpha internal consistency reliability coefficient ranged from 0.83 to 0.90, and test–retest correlation coefficient ranged from 0.68 to 0.73; it can be said that the internal consistency and test–retest reliability of the PTTUS are sufficient.

In recent years, internet usage has become widespread in Turkey and worldwide, and social media is one of the most concentrated areas of use. Social media allows people to interact with each other and share their opinions, thoughts, photos, and videos through applications such as YouTube, Facebook, Instagram, and TikTok. However, dependence on these applications can cause individual and interpersonal problems. The literature indicates that as addiction to social media applications increases, negative mood disorders such as anxiety, neuroticism, and depression and the time spent using social media increase (37–43). Some studies have shown that the relationship between addiction to social media applications and anxiety symptoms is higher than the relationship between symptoms of depression and anxiety (24, 25). It can be said that TikTok, which is mostly used by adolescents and young adults (16–35 years old), is likely to cause negative effects. Therefore, it is necessary to have a scale for measuring problematic TikTok use. In addition, it is recommended to develop interventions to prevent and reduce problematic TikTok use so that individuals experience less anxiety, depression, and other negative effects. Based on this information, PTTUS is a measurement tool with sufficient psychometric properties that can be applied to determine individuals’ levels of problematic use. The scale measures three sub-dimensions, and both the total score and the scores of the sub-dimensions can be obtained. Higher scores obtained from the scale indicate higher levels of problematic use.

The study was conducted online using a university population. Online data collection is being used increasingly, especially in social science. So large amounts of data were accessed quickly and at a low cost (44). Online data collection is as valid and reliable as traditional data collection methods. In addition to this strength, this study has several limitations. First, the participants were recruited using convenience sampling, so a more representative sample of the population is needed to generalize the findings. Second, data were obtained through self-report, so it may not be free from social desirability and recall biases. Future studies are needed to validate the PTTUS using an objective rating method rather than self-report. Third, the study was conducted in a non-clinical population. Further research is needed in clinical samples. Fourth, because this study only included participants in Turkey, there was no comparison of the PTTUS results between Western and Eastern countries, which could have important implications for healthcare professionals.

Nevertheless, this study provides initial support for using PTTUS as a reliable and valid measure of problematic TikTok use in Turkish adolescents and young adults. This easy-to-use scale has good psychometric properties and allows mental health professionals to screen for problematic TikTok use. It may also be useful for other researchers conducting studies related to problematic TikTok use in Turkey. Future studies are needed to demonstrate the usefulness of the scale for various age groups and problematic TikTok use behaviors in Türkiye.

## Data availability statement

The raw data supporting the conclusions of this article will be made available by the authors, without undue reservation.

## Ethics statement

The studies involving human participants were reviewed and approved by Ethics Committee: Pamukkale University, Social and Human Sciences Research and Publication Ethics Committee Document Decision Date and Number: 31.05.2022-E.211894. The patients/participants provided their written informed consent to participate in this study.

## Author contributions

AG, TO, and SC: conceptualization. AG and TO: data curation, formal analysis, and visualization. AG, TO, SY, and SC: methodology. All authors, writing—original draft and review and editing. All authors contributed to the article and approved the submitted version.

## Conflict of interest

The authors declare that the research was conducted in the absence of any commercial or financial relationships that could be construed as a potential conflict of interest.

## Publisher’s note

All claims expressed in this article are solely those of the authors and do not necessarily represent those of their affiliated organizations, or those of the publisher, the editors and the reviewers. Any product that may be evaluated in this article, or claim that may be made by its manufacturer, is not guaranteed or endorsed by the publisher.

## References

[1] WEARESOCIAL. Digital. Another year of bumper growth. Available online at: https://wearesocial.com/uk/blog/2022/01/digital-2022-another-year-of-bumper-growth-2/ (2022).

[2] Xiong Y Ji Y. From content platform to relationship platform: analysis of the attribute change of Tiktok short video (citation has been translated from Chinese7 language). View Publish. (2019) 4:29–34. doi: 10.54097/ehss.v5i.2901

[3] Wang Y. Humor and camera view on mobile short-form video apps influence user experience and technology-adoption intent, an example of TikTok (DouYin). Comput Hum Behav. (2020) 110:106373. doi: 10.1016/j.chb.2020.106373

[4] Montag C Yang H Elhai JD. On the psychology of TikTok use: a first glimpse from empirical findings. Front. Public Health. (2021) 9:641673. doi: 10.3389/fpubh.2021.641673, PMID: 33816425 PMC8010681

[5] Yang S Zhao Y Ma Y. Analysis of the reasons and development of short video application -taking TikTok as an example In: . 2019 9th international conference on information and social science (ICISS 2019). Francis Academic Press, UK (2019). 340–3. doi: 10.25236/iciss.2019.062

[6] Lodice R Papapicco C. To be a TikToker in COVID-19 era: an experience of social influence. Online J Comm Media Technol. (2021) 11:1–12. doi: 10.30935/ojcmt/9615

[7] Kawada T. Comment on "smartphone addiction proneness is associated with subjective-objective sleep discrepancy in patients with insomnia disorder". Psychiatry Investig. (2022) 19:595–6. doi: 10.30773/pi.2022.0024, PMID: 35903062 PMC9334804

[8] Kim SS Bae SM. Social anxiety and social networking service addiction proneness in university students: the mediating effects of experiential avoidance and interpersonal problems. Psychiatry Investig. (2022) 19:462–9. doi: 10.30773/pi.2021.0298, PMID: 35753685 PMC9233957

[9] Lim YJ. Exploratory structural equation modeling analysis of the social network site use motives scale. Psychiatry Investig. (2022) 19:146–53. doi: 10.30773/pi.2021.0092, PMID: 35164435 PMC8898605

[10] Shin NY. Psychometric properties of the Bergen social media addiction scale in Korean Young adults. Psychiatry Investig. (2022) 19:356–61. doi: 10.30773/pi.2021.0294, PMID: 35620820 PMC9136528

[11] Akin A Demirci I Kara S. The validity and reliability of Turkish version of the Facebook addiction scale. Academic perspective international refereed journal of. Soc Sci. (2017) 59:65–72.

[12] Kuss DJ Griffiths MD. Online social networking and addiction--a review of the psychological literature. Int J Environ Res Public Health. (2011) 8:3528–52. doi: 10.3390/ijerph8093528, PMID: 22016701 PMC3194102

[13] Turkyilmaz M. The translation of Facebook addiction scale into Turkish and impact of Facebook addition to reading ability. J Acad Soc Sci Stud. (2015) 6:265–80. doi: 10.9761/JASSS2942

[14] Kircaburun K. Effects of gender and personality differences on Twitter addiction among Turkish undergraduates. J Educ Pract. (2016) 6:265–42. doi: 10.9761/JASSS2942

[15] Moghavvemi SA Binti Sulaiman A I JN Kasem N. Facebook and YouTube addiction: the usage pattern of Malaysian students In: . 2017 international conference on research and innovation in information systems (ICRIIS). Langkawi, Malaysia (2017). 1–6. doi: 10.1109/ICRIIS.2017.8002516

[16] Tutgun-Ünal A. Social media addiction: A study on university students. İstanbul: Marmara University, Institute of Social Sciences (2015).

[17] Demirci I. The adaptation of the Bergen social media addiction scale to Turkish and its evaluation of relationships with depression and anxiety symptoms. Anatolian J Psychiatry. (2019) 20:1–22. doi: 10.5455/apd.41585

[18] Orbatu D Eliaçık K Alagayut D Hortu H Demirçelik Y Bolat N. Development of adolescent social media addiction scale: study of validity and reliability. Anatolian J Psychiatry. (202) 1:56–61. doi: 10.5455/apd.77273

[19] Özgenel M Canpolat Ö Ekşi H. Social media addiction scale for adolescents: validity and reliability study. Addicta. (2019) 0:629–62. doi: 10.15805/addicta.2019.6.3.0086

[20] Çam E. Educational and general purpose facebook uses and Facebook addictions of teacher candidates (SAU education faculty example). Sakarya: Sakarya University Institute of Educational Sciences (2012).

[21] Tabachnick BG Fidell LS. Using multivariate statistics. New York: Allyn and Bacon (2007).

[22] D’Souza L Samyukta A Bivera TJ. Development and validation of test for Instagram addiction (TIA). Int J Ind Psychol. (2018) 6:4–14. doi: 10.25215/0603.81

[23] Kavaklı M İnan E. Psychometric properties and correlates of the Turkish version of Instagram addiction scale (IAS). J Clin Psychol Res. (2021) 5:86–97. doi: 10.5455/kpd.26024438m000037

[24] Schou Andreassen C Billieux J Griffiths MD Kuss DJ Demetrovics Z Mazzoni E. The relationship between addictive use of social media and video games and symptoms of psychiatric disorders: a large-scale cross-sectional study. Psychol Addict Behav. (2016) 30:252–62. doi: 10.1037/adb0000160, PMID: 26999354

[25] van den Eijnden RJJM Lemmens JS Valkenburg PM. The social media disorder scale. Comput Hum Behav. (2016) 61:478–87. doi: 10.1016/j.chb.2016.03.038

[26] Sarıçam H Karduz FFA. The adaptation of the social media disorder scale to Turkish culture: validity and reliability study. Eğitimde ve Psikolojide Ölçme ve Değerlendirme Dergisi. (2018) 9:117–36. doi: 10.21031/epod.335607

[27] Filed A. Discovering statistics using SPSS. London: SAGE Publications Ltd. (2009).

[28] Çokluk Ö Şekercioğlu G Büyüköztürk Ş. Multivariate statistics for social sciences: SPSS and LISREL applications. Ankara: Pegem Academy Publishing (2014).

[29] Seçer İ. Practical data analysis with SPSS and LISREL. Ankara: Anı Publishing (2015).

[30] Brown TA. Confirmatory factor analysis for applied research. New York, US: Guilford Press (2006).

[31] Kline RB. Principles and practice of structural equation modeling. New York: The Guilford Press (1998).

[32] Schermelleh-Engel K Moosbrugger H Müller H. Evaluating the fit of structural equation models: tests of significance and descriptive goodness-of-fit measures. Methods of psychological research. Online. (2003) 8:23–74.

[33] Robinson JP Shaver PR Wrightsman LS. Criteria for scale selection and evaluation in measure of personality and social psychological attitudes. San Diego: California Academic Press (1991).

[34] Kuss D Griffiths M. Social networking sites and addiction: ten lessons learned. Int J Environ Res Public Health. (2017) 14:311–28. doi: 10.3390/ijerph14030311, PMID: 28304359 PMC5369147

[35] Pallant J. SPSS survival manual: A step by step guide to data analysis using SPSS for windows. Australia: Australian Copyright (2005).

[36] Büyüköztürk S. Data analysis handbook for social sciences. Ankara: Pegem Akademi Publishing (2008).

[37] Andreassen CS Torsheim T Brunborg GS Pallesen S. Development of a Facebook addiction scale. Psychol Rep. (2012) 110:501–17. doi: 10.2466/02.09.18.PR0.110.2.501-517 22662404

[38] Koc M Gulyagci S. Facebook addiction among Turkish college students: the role of psychological health, demographic, and usage characteristics. Cyberpsychol Behav Soc Netw. (2013) 16:297–84. doi: 10.1089/cyber.2012.0249 23286695

[39] Lin CY Brostrom A Nilsen P Griffiths MD Pakpour AH. Psychometric validation of the Persian Bergen social media addiction scale using classic test theory and Rasch models. J Behav Addict. (2017) 6:620–9. doi: 10.1556/2006.6.2017.071, PMID: 29130330 PMC6034942

[40] Lin CY Ganji M Pontes HM Imani V Brostrom A Griffiths MD. Psychometric evaluation of the Persian internet disorder scale among adolescents. J Behav Addict. (2018) 7:665–75. doi: 10.1556/2006.7.2018.88, PMID: 30264609 PMC6426385

[41] Pantic I Damjanovic A Todorovic J Topalovic D Bojovic-Jovic D Ristic S. Association between online social networking and depression in high school students: behavioral physiology viewpoint. Psychiatr Danub. (2012) 24:90–3. 22447092

[42] Uygur OF Uygur H Chung S Ahmed O Demiroz D Aydın EF. Validity and reliability of the Turkish version of the Glasgow sleep effort scale. Sleep Med. (2022) 98:144–51. doi: 10.1016/j.sleep.2022.06.022, PMID: 35853331

[43] Yang SC Tung CJ. Comparison of internet addicts and non-addicts in Taiwanese high school. Comp Human Behav. (2007) 23:79–96. doi: 10.1016/j.chb.2004.03.037

[44] Young KS. Cognitive behavior therapy with ınternet addicts: treatment outcomes and implications. CyberPsychol Behav. (2007) 10:671–9. doi: 10.1089/cpb.2007.9971, PMID: 17927535

