# Supplementary material for: Reliability and validity of the problematic TikTok Use Scale among the general population
Source: Front Psychiatry. 2023 Mar 28;14:1068431. doi: 10.3389/fpsyt.2023.1068431 (PMC10086164; doi:10.3389/fpsyt.2023.1068431)
Supplement: Supplementary file 1 [file Table_1.docx]

**Supplementary Table S1.** Findings Regarding the Criterion-Related Validity of the PTTUS

|  | BSMAS | SMD scale |
| --- | --- | --- |
| PTTUS Total | .56** | .49** |
| Obsession | .35** | .30** |
| Escapism | .52** | .39** |
| Lack of Control | .45** | .49** |

***p* <0.01

BSMAS, Bergen Social Media Addiction Scale; SMD, Social Media Disorder

**Supplementary Table S2. Cronbach’s Alpha Internal Consistency, McDonald’s Omega and Test-retest Reliability of the PTTUS**

|  | Number of Items | Cronbach’s Alpha | McDonald’s Omega | Test-retest  (Two-Week Intervals) |
| --- | --- | --- | --- | --- |
| PTTUS Total | 16 | .90 | .90 | .73** |
| Obsession | 4 | .83 | .84 | .68** |
| Escapism | 6 | .90 | .90 | .68** |
| Lack of Control | 6 | .85 | .85 | .70** |

***p* <.01

**Supplementary Table S3. The Results of Analysis Receiver Operating Characteristic (ROC) Based on Problematic Use**

| Status | AUC | CI 95% | Cut-off | Sensitivity | Specificity |
| --- | --- | --- | --- | --- | --- |
| Problematic user* | .91 | .87-.95 | 31.5 | .89 | .38 |

AUC, area under the curve; CI, confidence interval.

* Problematic use or addicted, excessive of social media and spending at least 8.5 to 21.5 hours a week online) are considered to indicate problematic use. Information was collected from participants about the time they spent using TikTok. These collected data were evaluated as problematic use in line with the social media addiction feature in the literature.
